# Creatine, Glutamine plus Glutamate, and Macromolecules Are Decreased in the Central White Matter of Premature Neonates around Term

**DOI:** 10.1371/journal.pone.0160990

**Published:** 2016-08-22

**Authors:** Meriam Koob, Angèle Viola, Yann Le Fur, Patrick Viout, Hélène Ratiney, Sylviane Confort-Gouny, Patrick J. Cozzone, Nadine Girard

**Affiliations:** 1 Service de Neuroradiologie, AP-HM Timone, Aix-Marseille Université, Marseille, France; 2 Service de Radiopédiatrie-Imagerie 2, CHU de Strasbourg, Hôpital de Hautepierre, Strasbourg, France; 3 Laboratoire ICube, UMR 7357, FMTS, Université de Strasbourg-CNRS, Strasbourg, France; 4 Aix-Marseille Université, CNRS, Centre de Résonance Magnétique Biologique et Médicale, UMR 7339, Faculté de Médecine la Timone, Marseille, France; 5 Laboratoire CREATIS, CNRS UMR 5220, Inserm U1044, Université Claude Bernard Lyon I, INSA-Lyon, Lyon, France; Linköping University, SWEDEN

## Abstract

Preterm birth represents a high risk of neurodevelopmental disabilities when associated with white-matter damage. Recent studies have reported cognitive deficits in children born preterm without brain injury on MRI at term-equivalent age. Understanding the microstructural and metabolic underpinnings of these deficits is essential for their early detection. Here, we used diffusion-weighted imaging and single-voxel ^1^H magnetic resonance spectroscopy (MRS) to compare brain maturation at term-equivalent age in premature neonates with no evidence of white matter injury on conventional MRI except diffuse excessive high-signal intensity, and normal term neonates. Thirty-two infants, 16 term neonates (mean post-conceptional age at scan: 39.8±1 weeks) and 16 premature neonates (mean gestational age at birth: 29.1±2 weeks, mean post-conceptional age at scan: 39.2±1 weeks) were investigated. The MRI/MRS protocol performed at 1.5T involved diffusion-weighted MRI and localized ^1^H-MRS with the Point RESolved Spectroscopy (PRESS) sequence. Preterm neonates showed significantly higher ADC values in the temporal white matter (*P*<0.05), the occipital white matter (*P*<0.005) and the thalamus (*P*<0.05). The proton spectrum of the centrum semiovale was characterized by significantly lower taurine/H_2_O and macromolecules/H_2_O ratios (*P*<0.05) at a TE of 30 ms, and reduced (creatine+phosphocreatine)/H_2_O and (glutamine+glutamate)/H_2_O ratios (*P*<0.05) at a TE of 135 ms in the preterm neonates than in full-term neonates. Our findings indicate that premature neonates with normal conventional MRI present a delay in brain maturation affecting the white matter and the thalamus. Their brain metabolic profile is characterized by lower levels of creatine, glutamine plus glutamate, and macromolecules in the centrum semiovale, a finding suggesting altered energy metabolism and protein synthesis.

## Introduction

Preterm birth represents a greater risk of periventricular white matter injury, a myelin pathology often associated with neuroaxonal damage involving both the cortex and the subcortical nuclei [1]. These lesions are considered responsible for various degrees of motor, sensory and cognitive deficits. Over the past decade, the incidence of cystic periventricular leukomalacia (PVL) has decreased, whereas diffuse white matter injuries including diffuse excessive high-signal intensity (DEHSI) and punctuate white matter lesions have become more frequent [2–4]. This evolution toward lesions thought to represent milder forms of PVL [2] resulted in a net decrease of severe motor disabilities, but a persistence of cognitive and behavioral disorders generally detected in adolescent and adult life.

The risk of cognitive deficit has been correlated with the presence and severity of white matter lesions on conventional MRI at term-equivalent age [5–8], whereas normal conventional MRI is viewed as an indicator of favorable neurodevelopmental outcome [6, 7]. However, several studies have reported cognitive impairment in children born prematurely despite normal findings on conventional MRI at term-equivalent age [9–11]. The neural correlates underlying later cognitive impairment could be linked to subtle developmental brain anomalies undetectable with conventional MRI.

Many advanced neuroimaging techniques and image analysis tools have been used to investigate brain development in preterm neonates, including diffusion-weighted imaging and diffusion tensor imaging, functional MRI, mathematical methods for cortical morphometric analysis and MR spectroscopy [12]. Diffusion-weighted imaging and diffusion tensor imaging are well suited for the study of white matter architecture and organization and have demonstrated delayed white matter maturation in preterm brains compared to term brains [4, 13–15]. ADC was found increased in the centrum semiovale of preterm neonates, a finding correlated to lower cognitive function at 2 years of age [16]. Recently, MRI-based tractography studies have identified alterations of structural connectivity in preterm infants without white matter injury at term-equivalent age [17, 18]. Volume reduction in specific structures and altered cortical thickness/sulcation were found in preterm neonates imaged at term-equivalent age [19, 20]. These abnormalities persisted in adolescents and adults [21] and were correlated to cognitive impairment [9, 22, 23].

Alterations of brain metabolism in preterm neonates with mild to severe brain injury have been investigated with protons magnetic resonance spectroscopy (^1^H-MRS) around term. Recent studies performed at short time of echo (TE = 35 ms) reported abnormal *N-*acetylaspartate to choline-containing compounds (NAA/Cho) ratio in the cortex and the subventricular zone in preterms born with extreme low birth weight [24], and reduced NAA in the parietal white matter of preterm neonates with punctuate white matter lesions [25]. These findings point to neuronal damage and myelin injury. Only few MRS studies comparing the brain metabolic profile of term and premature neonates with no evidence of white matter damage on conventional MRI except DEHSI have been carried out at term-equivalent age. In two studies performed at short echo times (TE of 7 ms or 20 ms), no significant difference was observed in the precentral area [26] the thalamus, the gray and the white matter, except a non significant trend for higher creatine concentration in preterm neonates [27]. Low *myo*-inositol (*myo*-Ins) concentrations were found in the parietal white matter of preterm with DEHSI in comparison to term neonates, a finding suggesting possible astrogliosis [25]. In another study, γ-amminobutyric acid (GABA) edited-spectra obtained with the MEshcher-GArwood Point RESolved Spectroscopy (MEGA-PRESS) sequence (TE of 68 ms) showed decreased GABA and glutamate levels in the frontal region in the preterm group [28]. Bluml *et al*. found increased levels of NAA and creatine in the parietal white matter of preterm neonates (TE of 35 ms) aged 270 to 370 post-conceptional days in comparison to age-matched term neonates [29]. The apparent discrepancy between these results could be linked to the small size of some cohorts, the difficulty to have the same age range around term in preterm and term groups, and regional differences in maturation and vulnerability to prematurity.

The purpose of this study was to assess brain maturation at term-equivalent age in preterm neonates without overt lesions on conventional MRI, except DEHSI, and with normal neurological outcome at 2 years of age, and in full-term neonates using diffusion-weighted MRI and ^1^H-MRS. We selected premature and full-term neonates with comparable age at scan (premature neonates: 39.8±1 weeks post-conceptional age (PCA), full-term neonates: 39.2±1 weeks PCA). We hypothesized that some metabolic anomalies could have gone undetected at short echo time, since metabolite T_2_, and particularly the T_2_ of choline-containing compounds had been reported to be shorter in the premature brain around term [30]. We therefore analyzed ^1^H-MRS performed with the PRESS sequence at short and long TEs (30 and 135 ms). Because we assumed that prematurity could have an impact on lipid and protein synthesis, we used a time-domain quantitation algorithm based on semi-parametric Quantum ESTimation (QUEST) and a simulated metabolic database allowing the analysis of metabolites and macromolecules including mobile lipids. Our results suggest that metabolite and macromolecules synthesis may be reduced in the brain of preterm neonates at term-equivalent age.

## Subjects and Methods

### Ethics statement

Verbal or written consent was not necessary because the MRI/MRS protocol was performed in a clinical setting with the standard MRI/MRS sequences used in our institution (including diffusion-weighted MRI and ^1^H-MRS) for the clinical examination of all neonates whether premature or not. The MRI/MRS data were anonymized by Pr Nadine Girard (head of the department of Neuroradiology at La Timone Hospital) after collection, before they were transferred to the research database at the Center for Magnetic Resonance in Medicine and Biology for analysis. This anonymization procedure and the retrospective analysis of the data were approved by our Institutional Ethics Committee (Comité d’Ethique d’Aix-Marseille Université, project number n° 2014-09-30-07).

### Subjects

MRI/MRS explorations of neonates conducted in our institution over a two-year time period were reviewed for this retrospective study. Premature neonates were imaged as part of the routine morphological evaluation before discharge from hospital near term, whereas term neonates were explored in the context of secondary workup to rule out brain malformation, congenital infection and stroke and to verify mild ventriculomegaly diagnosed *in utero*.

Only non-anesthetized non-sedated subjects were selected for this study. The first step of the selection process consisted in identifying morphologically and clinically normal premature neonates, born with an appropriate weight for age at birth, with no evidence of cortical atrophy, cystic PVL and punctate WML on conventional MRI, although DEHSI with visible cross-roads could be present [31]. Only term-neonates with brain MRI considered morphologically normal with no evidence of cortical atrophy were chosen. Cases with clinical evidence of maternofetal infection, neonatal infection, metabolic disease, and birth asphyxia were excluded from the study as well as those with germinal matrix hemorrhage, ventriculomegaly or hydrocephalus. Term neonates with a suspicion of maternofetal infection, which were found negative, were included in the study provided the morphology was normal.

The second step of the selection process consisted in identifying among all previously selected neonates explored with both diffusion-weighted MRI and ^1^H-MRS, those examined around term (38 weeks ≤ post-conceptional age ≤ 42 weeks). Among them, only those showing no sign of motion artifact on both conventional MRI and diffusion-weighted MRI, and no misplaced region of interest (ROI) for white-matter spectroscopy were retained. Finally, thirty-two subjects were selected, 16 term neonates (TN) (mean PCA at scan: 39.8±1 weeks) and 16 premature neonates (PN) (mean gestational age (GA) at birth: 29.1±2 weeks, mean PCA at scan: 39.2±1 weeks). Among the 16 PN neonates, 8 had DEHSI on conventional MRI. The statistical comparison of the ages at MRI using the Mann-Whitney test did not show any significant difference between both groups (*P* = 0.1414).

### MRI and MRS protocol

MR examinations were performed on a clinical MR system operating at 1.5 Tesla (Symphony Maestro Class, Siemens, Germany).

#### MRI protocol

Neonates were explored without sedation or general anesthesia. An eight-channel head coil was used for these examinations. The standard MRI protocol included half-Fourier acquisition single-shot turbo spin-echo images and gradient-echo T_1_-weighted images following the 3 planes of the neonatal head. Diffusion-weighted MRI was performed with axial multi-slice multi-repetition spin-echo echo-planar technique (time of repetition = 3200 ms, TE = 102 ms, slice thickness = 4 mm, three averages per image, field of view = 240x240 mm^2^, acquisition matrix 128x128). Diffusion was measured in 3 orthogonal directions from 3 values of b (b = 0, 500, and 1000 s/mm^2^).

#### Diffusion-MRI data processing

**Fig 1:**
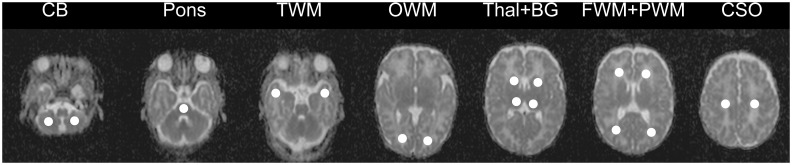
Position of regions of interest (ROIs) on cerebral ADC maps.

Diffusion-weighted MRI and the averaged apparent diffusion coefficient (ADC) maps were automatically generated using Siemens software. Circular single-sized ROIs of 5 mm radius were carefully placed to avoid contamination from adjacent cerebrospinal fluid. ADC values were measured in the cerebellar white matter, the central pons, the white matter of the temporal pole, the occipital white matter of the calcarine area, the median area of the thalamus, the basal ganglia, the frontal white-matter (in front of the frontal horns approximately at the level of the F1-F2 junction), the parietal white-matter at the level of the atria (PWM), and the centrum semiovale (at the level of the white matter underlying the central sulcus) (Fig 1). Bilateral ROIs were positioned for all brain structures except central pontine white matter (one single ROI).

The ADC maps were obtained from a preterm neonate (GA at birth: 32 weeks, PCA at MRI: 40 weeks). Location of the ROIs from left to right: ADC values were measured in the cerebellar white matter (CB), the central pons, the white matter of the temporal pole (TWM), the occipital white matter of the calcarine area (OWM), the median area of the thalamus (Thal), the basal ganglia (BG), the frontal white-matter (in front of the frontal horns approximately at the level of the F1-F2 junction) (FWM), the parietal white-matter at level of the atria (PWM), and the centrum semiovale (at the level of the white matter underlying the central sulcus). Bilateral ROIs were positioned for all brain structures except central pontine white matter (one single ROI).

#### MRS protocol

The volume of interest for spectroscopy was centered at the level of the white matter underlying the central sulcus within the centrum semiovale. Localized ^1^H-MRS was performed using a PRESS sequence (time of repetition = 1500 ms, TE = 30 and 135 ms, voxel size of 4.5 cm^3^, 256 averages for TE = 30 ms and 278 averages for TE = 135 ms). ^1^H-MRS spectra were acquired with water saturation with the Chemical Shift-Selective module at both TEs. An additional spectrum was acquired at a TE of 30 ms without water saturation.

#### Proton-MRS data processing

MRS data were processed under CSIAPO, a home-made software [32] using a time-domain quantitation algorithm based on QUEST [33] and a simulated metabolic database created with VESPA and the GAVA package (http://scion.duhs.duke.edu/vespa/). The metabolite basis set included 15 metabolites (alanine, aspartate, β-glucose (resonances arising between 3.2 and 3.9 ppm), phosphocholine, creatine, glutamine, glutamate, glycine, glycerophosphocholine, lactate, *myo*-Ins, NAA, phosphocreatine, *scyllo-*inositol and taurine), and 12 different macromolecular resonances (MM_9, MM_12, MM_14, MM_17, MM_19, MM_20, MM_22, MM_27, MM_30, MM_32, MM_37, and MM_39). MM_9 and MM_12 correspond both to the resonances of methylene and methyl groups of mobile lipids, and to the resonances of aminoacids of mobile polypeptide chains. These MM components were determined from the fit of a macromolecule spectrum obtained using a separate inversion recovery acquisition. During the fitting procedure, each MM component was adjusted according to constraints applied on their amplitudes (A_MMi_) and frequency shifts (f_MMi_) and expressed as follows: A_MMi_ = *ri*, A_MM_9_ with 0.6≤ *ri* ≤1.4 and f_MMi_ = f_MM_9_ ± 8Hz, MM_9 corresponding to the resonating group at 0.9 ppm, and i designating the eleven other resonances [34, 35]. These constraints were chosen to allow little flexibility in the macromolecular modeling, while the relative amplitudes in the basis set respected the ones found in the inversion recovery experiment. Alanine, aspartate, β-glucose and macromolecules were not fitted on spectra acquired at long echo time. The signals of metabolites were normalized using water signal intensity as an internal reference [36]. The results obtained at short and long TE were expressed as ratios of the relative area of each metabolite signal to the water signal obtained at a TE of 30 ms to avoid a T_2_ effect on this internal reference. Several signals were pooled together because the resolution of spectra at 1.5 T did not permit to fit them independently: creatine plus phosphocreatine corresponding to total creatine (tCr), phosphocholine plus glycerophosphocholine corresponding to total choline (tCho), glutamine plus glutamate (Glx) and *myo*-inositol plus glycine (*myo*-Ins+Gly). Because we cannot exclude the possibility that NAA signal may include *N*-acetyl-aspartyl-glutamate contribution, it was referred to as total NAA (tNAA). Metabolite transverse relaxation times (T_2_) were determined assuming a monoexponential decay of T_2_ relaxation and a metabolite intensity (metabolite/H_2_O) of zero when TE tends towards ∞. Water was used as an internal reference to normalize metabolite signal intensities by correcting B1 inhomogeneities and partial volume effects. Consequently, metabolite/H_2_O ratios were used to generate curves of metabolite signal intensities as a function of TE and calculate metabolite T_2_ relaxation times using the following equation:

$${\text{Y}=\text{Y}}_{\text{0}}\times e^{\left(-\frac{TE}{T2}\right)}$$

with Y = metabolite_(TE)/_H_2_O_(TE = 30ms)_, Y_0 =_ metabolite_(TE = 0 ms)_/H_2_O_(TE = 30ms)_

Although the use of only two TEs is not optimal, and despite the absence of a very long TE for the evaluation of long T_2_ (i.e. T_2_ of tNAA, tCho or lactate), this model allowed the calculation of an apparent T_2_ for metabolites known or supposed to have short T_2_ (T_2_<250 ms) namely tCr, Glx, taurine and *myo*-Ins+Gly [30, 37–41]. The exponential regression was applied to each group dataset and not to each individual because there were too few data points available in this later case. Consequently, the T_2_ values obtained for a given metabolite with the exponential fitting procedure were group values. The fitting algorithm was based on the least squares methods and the goodness of the fit was quantified by the coefficient of determination R^2^. With this method based on group values [42] it was not possible to statistically compare the two values obtained after the exponential fitting procedure for each metabolite [42].

### Statistical analysis

A non-parametric analysis was used. ADC values obtained from various ROIs of the left and right hemispheres were compared using the Wilcoxon test. The comparison of ADC values and metabolic ratios between both groups of subjects for each brain region was performed using the Mann-Whitney test. The non-parametric Spearman correlation test was used to analyze dependence between metabolite or macromolecule ratios and age. Statistical analysis was performed using GraphPad Prism version 5.00 (San Diego, CA) and JMP SAS version 9.0.0 (Cary, NC). Values are reported as means ± standard deviation (sd) or standard error of the mean (sem). *P* values < 0.05 were considered significant.

## Results

### Brain ADC values are higher in the thalamus, the temporal white-matter and the occipital white-matter of preterm neonates at term-equivalent age

Diffusion-weighted images and ADC maps were obtained for 15 PN and 15 TN in 9 regions of interest (Fig 1). No statistical difference was observed between the right and left hemispheres for ADC values in symmetrical ROIs in PN and TN (Table 1). Consequently, the left and right values obtained for each ROI were averaged before group comparison. Since ADC values in PN with DEHSI were not statistically different from those measured for PN without DEHSI (Table 1), the all group of PN was used for further comparison with the TN group. ADC values were found higher in 7 ROIs out of 9 in PN. ADC values were significantly higher in 3 regions, the thalamus (+ 4%, P< 0.05), temporal white matter (+7%, P<0.05), and occipital white matter (+12%, P< 0.005) (Table 1).

**Table 1:** Comparison of ADC values between PN and TN.

	ADC (x10^-6^ mm^2^ /s)				
Cerebral structures	TN (n = 15)	PN_tot_ (n = 15)	PN with DEHSI (n = 8)	PN without DEHSI (n = 7)	Mann-Whitney test
Cerebellum	120.22±8.3	113.61±8.52	113.59±9.43	113.63±8.31	*PN*^*+*^ *vs PN*^*−*^ *P = 0*.*9551*
					*TN vs PN*_*tot*_ *P = 0*.*0512*
Pons	98.52±6.98	96.59±5.05	97.06±6.36	96.18±3.99	*PN*^*+*^ *vs PN*^*−*^ *P = 0*.*7789*
					*TN vs PN*_*tot*_ *P = 0*.*5615*
Basal ganglia	114.06±7.74	117.99±5.28	117.44±5.89	118.48±5.03	*PN*^*+*^ *vs PN*^*−*^ *P = 0*.*5621*
					*TN vs PN*_*tot*_ *P = 0*.*0537*
Thalamus	103.98±9.10	108.26±4.13	107.35±4.69	109.05±3.70	*PN*^*+*^ *vs PN*^*−*^ *P = 0*.*4634*
					***TN vs PN***_***tot***_ ***P = 0*.*0213***
Temporal white matter	146.46±14.84	156.33±5.82	155.57±6.64	157.00±5.37	*PN*^*+*^ *vs PN*^*−*^ *P = 0*.*8665*
					***TN vs PN***_***tot***_ ***P = 0*.*0380***
Occipital white matter	138.81±14.27	155.35±8.04	155.99±9.14	154.79±7.56	*PN*^*+*^ *vs PN*^*−*^ *P = 0*.*9551*
					***TN vs PN***_***tot***_ ***P = 0*.*0016***
Frontal white matter	167.31±14.28	177.18±13.36	180.55±16.63	174.225±9.93	*PN*^*+*^ *vs PN*^*−*^ *P = 0*.*8665*
					*TN vs PN*_*tot*_ *P = 0*.*1057*
Parietal white matter	166.85±7.66	174.80±13.89	180.97±15.37	169.406±10.60	*PN*^*+*^ *vs PN*^*−*^ *P = 0*.*1520*
					*TN vs PN*_*tot*_ *P = 0*.*0620*
Centrum semiovale	134.24±16.09	139.53±11.82	136.475±10.22	143.01±13.33	*PN*^*+*^ *vs PN*^*−*^ *P = 0*.*6954*
					*TN vs PN*_*tot*_ *P = 0*.*3195*

Abbreviations: ADC = apparent diffusion coefficient; PN = premature neonates; PN_tot_ = PN with DEHSI + PN without DEHSI; TN = term neonates.

### Brain levels of creatine, Glx, and taurine are lower in preterm neonates at term-equivalent age

**Fig 2:**
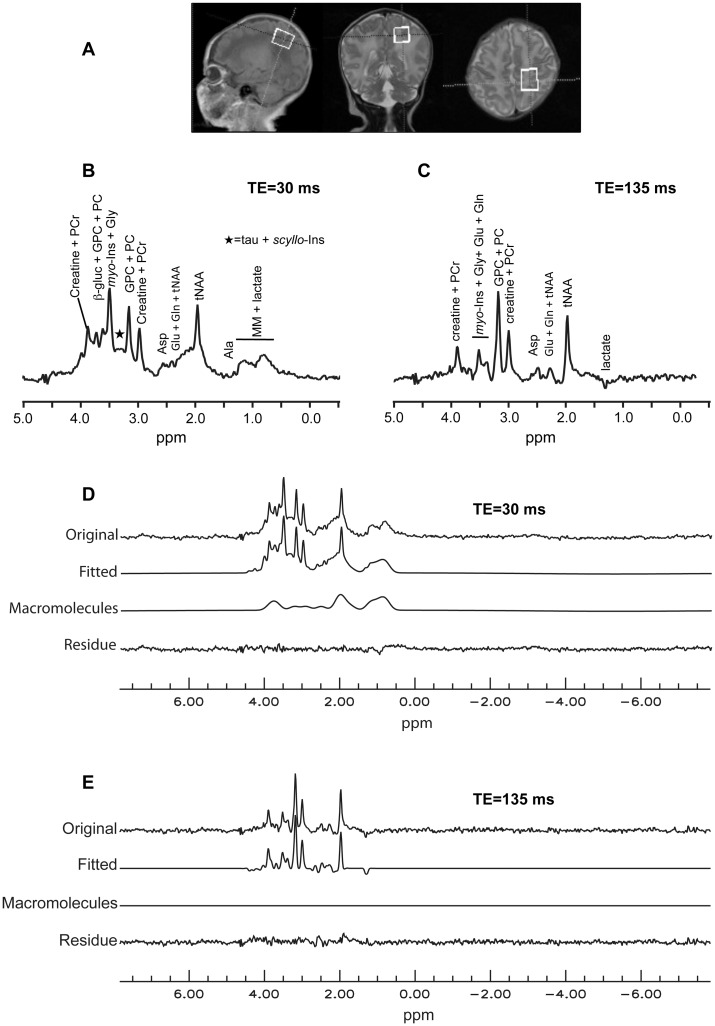
Typical ^1^H-MRS spectra obtained in the centrum semi ovale of a term neonate. The spectra were obtained from a term neonate born at 39 weeks GA and imaged at 41 weeks PCA. **A:** Localization of the voxel. **B and C:** ^1^H-MRS spectra obtained at short (30 ms) and long TE (135 ms). **D and E:** Spectra processing with QUEST. A global fitted spectrum including metabolites and macromolecules was generated (“Fitted”). A second fitted spectrum corresponding to macromolecules signals only (“Macromolecules”) was produced and subtracted from the global fitted spectrum. The residue shows residual metabolites signals after subtraction of fitted metabolites and macromolecules signals. Note the absence of detectable macromolecule signals on the spectrum obtained with a TE of 135 ms. **Abbreviations:** Ala: alanine, Asp: aspartate, β-gluc: β-glucose, Glu: glutamate, Gln: glutamine, Gly: glycine, GPC: glycerophosphocholine, *myo*-Ins: *myo*-inositol, tNAA: total *N*-acetylaspartate, PC: phosphocholine, PCr: phosphocreatine, *scyllo*-Ins: *scyllo*-inositol, tau: taurine.

**Fig 3:**
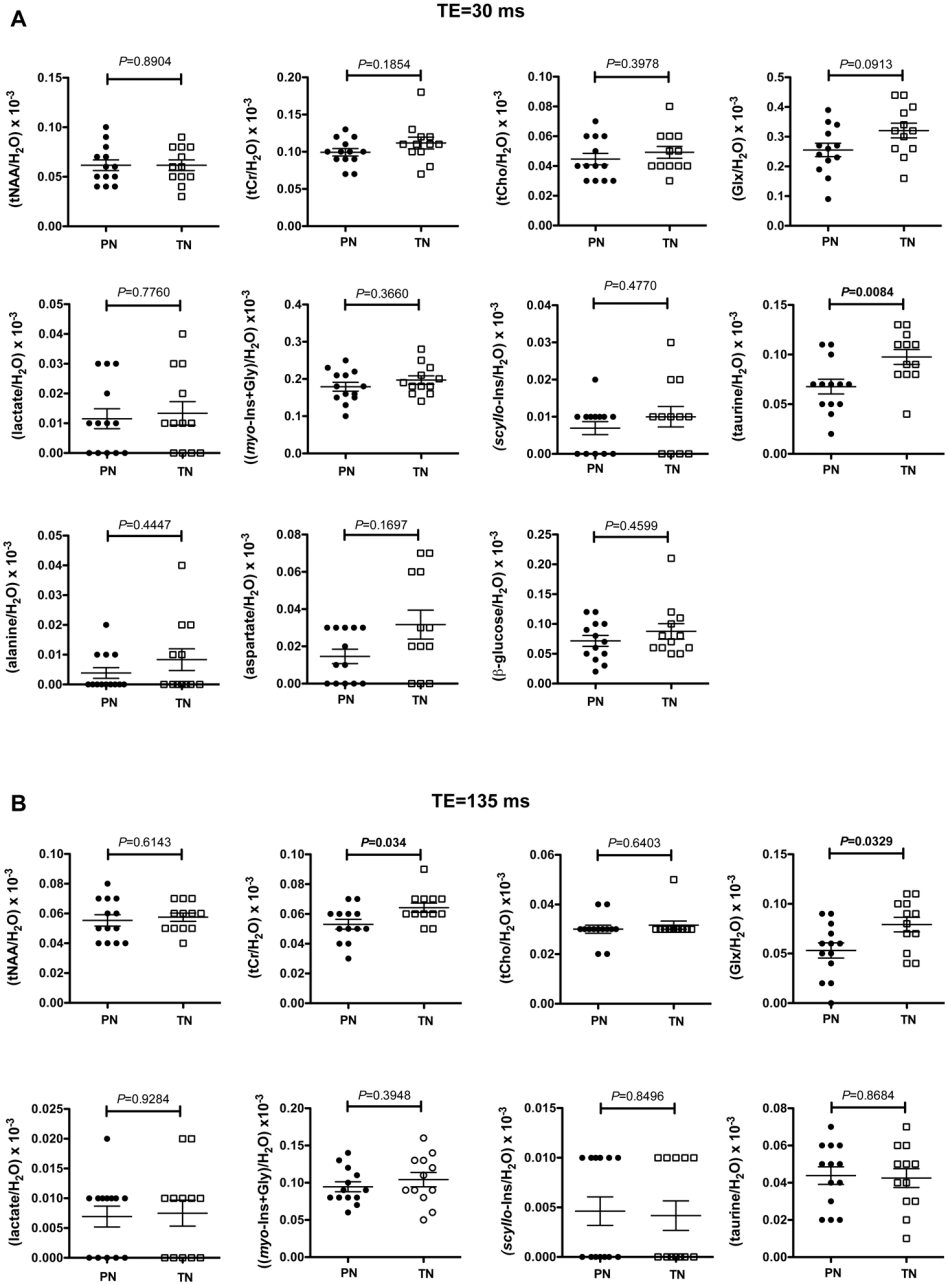
Variations of metabolic ratios between premature and term brains around term. **A:** Metabolic ratios from spectra obtained with a TE of 30 ms. **B:** Metabolic ratios from spectra obtained with a TE of 135 ms. **C:** Assessment of the T_2_ effect on main metabolic signals. The ratio of metabolic signals obtained at 30 and 135 ms was calculated for tNAA, Cho, tCr and Glx. **Abbreviations:** tCho: glycerophosphocholine+phosphocholine, tCr: total creatine (creatine+phosphocreatine), Glx: glutamate+glutamine, *myo*-Ins: *myo*-inositol, tNAA: total *N*-acetylaspartate, PN: premature neonates, TN: term neonates. Values are expressed as means ± sem.

Good quality spectra in terms of signal to noise ratio (SNR) and linewidth were obtained for 13 PN and 12 TN. The resonance signal linewidths reported by CSIAPO software for tCho signal from water-suppressed spectra were comprised between 2 and 5 Hz for spectra acquired with a TE of 30 ms, and between 3 and 7 Hz for those acquired with a TE of 135 ms. The SNR was superior to 14 for spectra acquired with a TE of 30 ms and to 10 for those acquired with a TE of 135 ms. Fig 2A–2E shows typical spectra obtained at short and long echo times and the corresponding fitted spectra. At a TE of 30 ms, Glx/H_2_O tended to be lower in PN (-20%), but the only significant difference between PN and TN was the higher taurine/H_2_O ratio in TN (+30%) (Fig 3A). At a longer TE (135 ms), significant variations of tCr/H_2_O and Glx/H_2_O were observed between both groups. These metabolic ratios were significantly lower in premature neonates than in term neonates (-17% for tCr/H_2_O and -33% for Glx/H_2_O) (Fig 3B).

**Fig 4:**
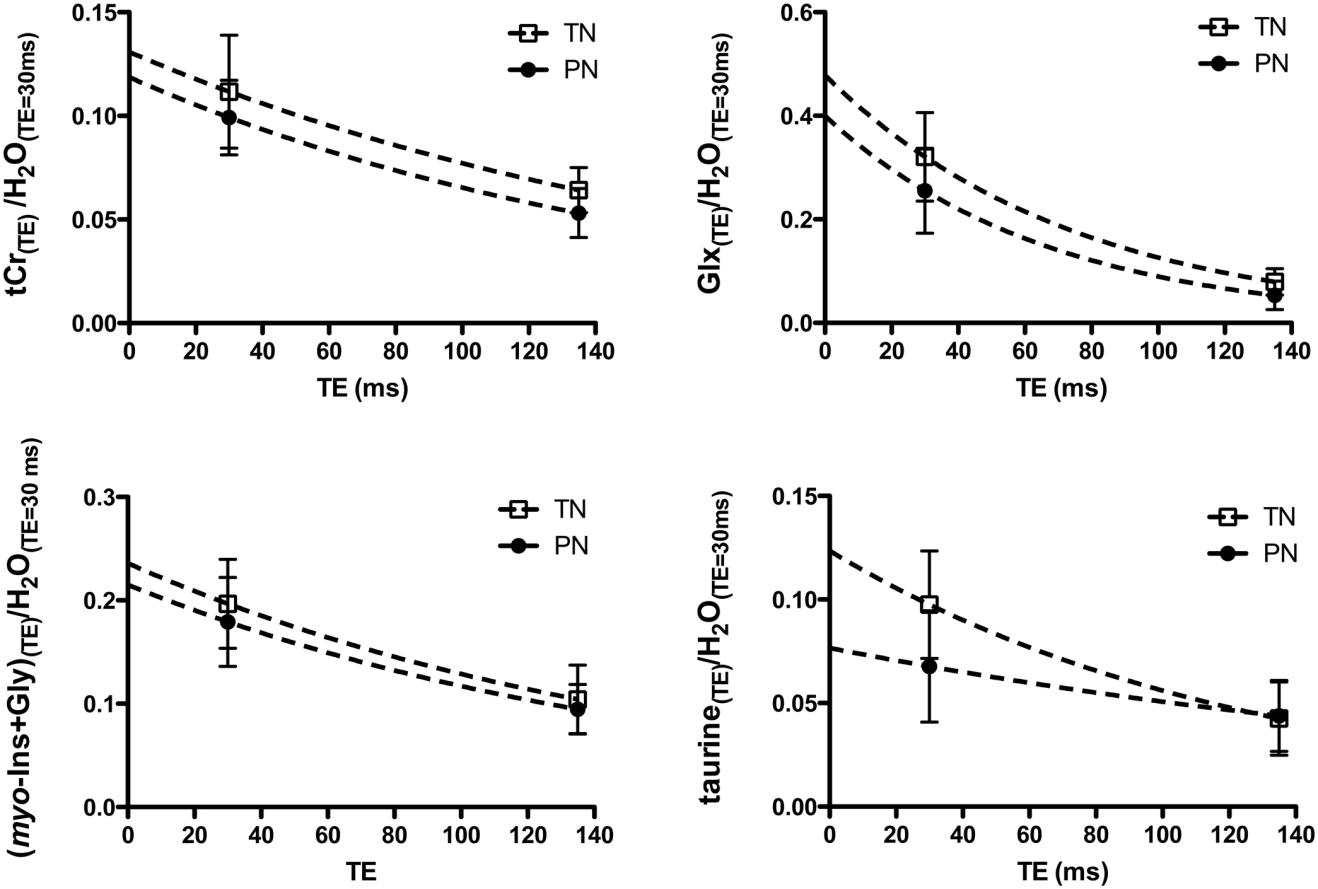
Monoexponential regression of metabolite signal intensity ratios as a function of TE for apparent T_2_ calculation. Metabolite signal intensity (metabolite/H_2_O) was fitted with a monoexponential decay model with a plateau constrained to a value of 0. Values are expressed as means ± sd.

To determine whether these variations were linked to differences in metabolite T_2_ relaxation time and/or concentration, T_2_ relaxation times of tCr, Glx, taurine and *myo*-Ins+Gly were estimated using a monoexponential regression model (Fig 4, Table 2). The goodness of the fit was quantified by the coefficient of determination R^2^. The shorter the T_2_, the greater were the R^2^ values (Table 2). The lowest R^2^ value was obtained for taurine in PN (apparent T_2_ = 242 ± 94 ms, R^2^ = 0.2334). Apparent T_2_ values measured for tCr and (*myo*-Ins+Gly) in PN and TN were in the same range of values measured at 1.5 T with the PRESS sequence in the basal ganglia of premature brain at 37.8 ± 2.2 weeks PCA [30] (tCr = 228 ms ± 12%, *myo*-Ins = 173 ms ± 67%). To our knowledge, T_2_ values of Glx and taurine have not been previously measured in the neonatal brain at 1.5 T. Our T_2_ values of Glx were twice lower than published values measured in the adult brain white matter at 3T [39], whereas the only comparative T_2_ values available for taurine were obtained in the adult human brain at 7T and varied from 85 ± 10 to 120 ± 20 ms [41]. PN exhibited lower apparent T_2_ values for almost all metabolites (Glx, tCr and (*myo*-Ins+Gly)) except for taurine. In this case, the apparent T_2_ was increased by 91.15% (Table 2). Y_0_, which represents the signal intensity ratio when signal loss due T_2_ effect is the lowest, was generally reduced in PN. The largest Y_0_ variation was observed for taurine (-37.99%). The metabolite signal intensity ratios significantly reduced at long TE in PN (tCr/H_2_O and Glx/H_2_O) (Fig 3) corresponded to lower Y_0_ and apparent T_2_ in PN than in TN.

**Table 2:** Apparent T_2_ relaxation time estimates.

Parameters	tCr	Glx	taurine	*(myo*-Ins+Gly)
**Apparent T**_**2**_ **(TN)** ^(a)^	189 ± 37 ms	75 ± 13 ms	126.5 ± 25 ms	165.3 ± 31 ms
	(± 19.42%)	(± 16.93%)	(± 19.82%)	(± 18.94%)
**Apparent T**_**2**_ **(PN)** ^(a)^	168 ± 24 ms	67 ± 14 ms	242 ± 94 ms	164.4 ± 29.71 ms
	(± 14.43%)	(± 20.76%)	(± 39.02%)	(± 18.07%)
**Y**_**0**_ **(TN)** ^(a)^	0.131 ± 9.67*10^−3^	0.478 ± 4.70*10^−2^	0.124 ± 1.17*10^−2^	0.236 ± 1.85*10^−2^
**Y**_**0**_ **(PN)** ^(a)^	0.119 ± 7.03*10^−3^	0.400 ± 5.00*10^−2^	0.077 ± 9.60*10^−3^	0.215 ± 1.62*10^−2^
**R**^**2**^ **(TN)**	0.5889	0.8	0.6259	0.6129
**R**^**2**^ **(PN)**	0.7132	0.7481	0.2334	0.6422
**[Y**_**0**_ **(PN)/Y**_**0**_ (TN)-1]*100	-9.32%	-16.38%	-37.99%	-8.82%
**[T**_**2**_ **(PN)/T**_**2**_ (TN)-1]*100	-9.23%	-13.88%	+91.15%	-0.54%

^(a)^ = mean ± SD,

Y0 = metabolite_(TE)_/H_2_0_(TE = 30 ms)_.

Sd for apparent T_2_ relaxation times are expressed in ms and in percentage.

The Spearman correlation test was applied to analyze the possible dependence between metabolite ratios and gestational age at birth or post-conceptional age. Metabolite ratios, which were found significantly different between PN and TN groups, were selected for this analysis. tCr/H_2_O tended to be correlated with gestational age at birth, although not reaching statistical significance (ρ = 0.3439, *P =* 0.0923), and appeared independent of post-conceptional age (ρ = 0.1991, *P =* 0.3399). No significant correlation was measured for the Glx/H_2_O ratio with either PCA (ρ = 0.1991, *P =* 0.3399) or GPA (ρ = 0.1601, P = 0.2896) whereas tau/H_2_O was significantly correlated to gestational age at birth (ρ = 0.5275, *P =* 0.0067) but was independent of post-conceptional age (ρ = 0.2101, *P =* 0.3135).

### MM_27, MM_37 and MM_39 are lower in preterm neonates at term-equivalent age and full-term neonates

**Fig 5:**
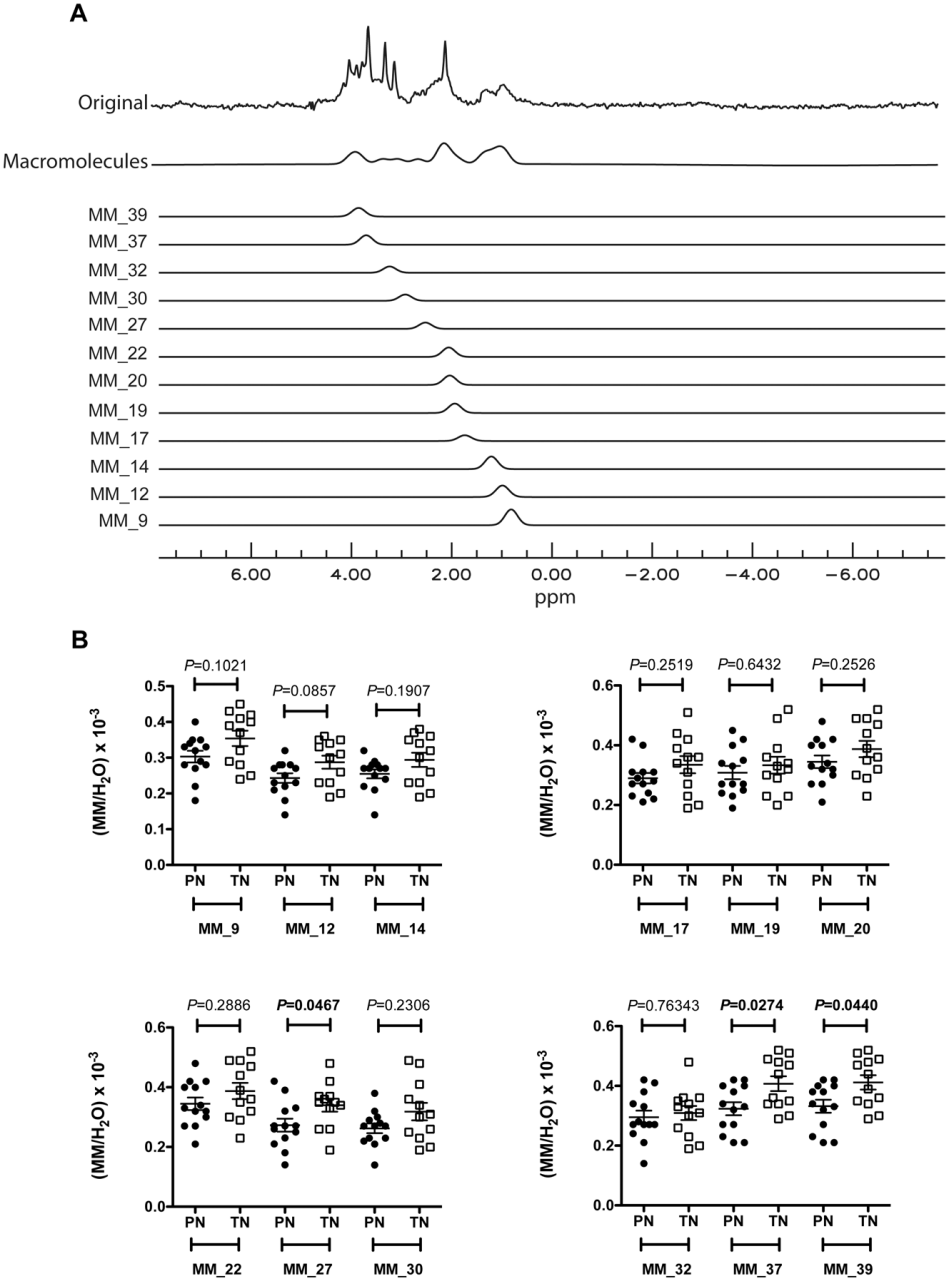
Variations of macromolecule ratios between premature and term brains around term. **A:** Macromolecule signals processing with QUEST for spectra obtained with a TE of 30 ms. The fitted spectrum of all macromolecule signals (“Macromolecules”) is the sum of 12 different fitted macromolecules: MM_9 (δ_ppm_ = 0.9), MM_12 (δ_ppm_ = 1.2), MM_14 (δ_ppm_ = 1.4), MM_17 (δ_ppm_ = 1.7), MM_19 (δ_ppm_ = 1.9), MM_20 (δ_ppm_ = 2), MM_22 (δ_ppm_ = 2.2), MM_27 (δ_ppm_ = 2.7), MM_30 (δ_ppm_ = 3), MM_32 (δ_ppm_ = 3.2), MM_37 (δ_ppm_ = 3.7) and MM_39 (δ_ppm_ = 3.9). **B**: Variations in macromolecule ratios between premature and term neonates around term. **Abbreviations:** MM: macromolecules, PN: premature neonates. TN: term neonates. Values are expressed as means ± sem.

**Fig 6:**
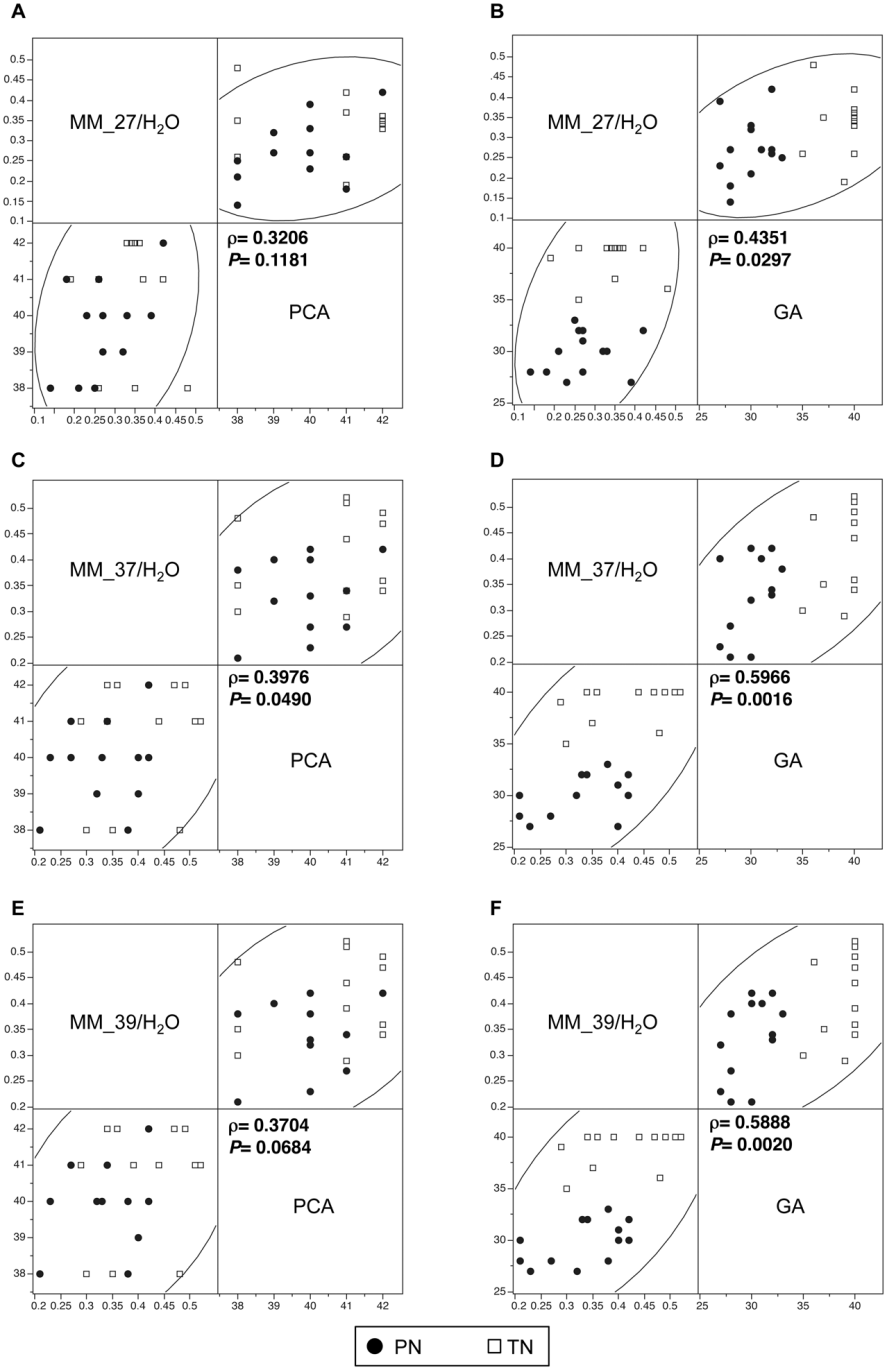
Correlations between macromolecule ratios and post-conceptional age or gestational age. The results of the Spearman’s correlation test between macromolecule ratios and GA or PCA are shown only for ratios significantly different between PN and TN (MM_27/H_2_O, MM_37/H_2_O and MM_39/H_2_O for a TE of 135 ms). **A and B:** MM_27/H_2_O variations as a function of post-conceptional age **(A)** and of gestational age **(B). C and D:** MM_37/H_2_O variations as a function of post-conceptional age **(C)** and of gestational age **(D)**. **E and F:** MM_39/H_2_O variations as a function of post-conceptional age **(E)** and of gestational age **(F). Abbreviations:** GA: gestational age, PCA: post-conceptional age, PN: premature neonates, TN: term neonates.

The analysis of ^1^H spectra obtained from the centrum semiovale at short TE showed a difference in the macromolecule content between PN and TN (Fig 5). There was a trend for MM_12/H_2_O to be lower in the PN group (Fig 5B), whereas MM_27/H_2_O, MM_37/H_2_O and MM_39/H_2_O were significantly lower in PN than in TN (Fig 5B). The non-parametric Spearman correlation test was used to search for a possible dependence between macromolecule ratios and gestational age at birth or post-conceptional age (Fig 6A–6F). The macromolecule ratios, which were significantly different in PN and TN groups, were selected for this analysis. **A** significant positive correlation was found for MM MM_37/H_2_O with post-conceptional age, while there was a trend for MM_39/H_2_O, although not reaching statistical significance (Fig 6A, 6C and 6E). MM_27/H_2_O, MM_37/H_2_O and MM_39/H_2_O were also positively correlated to gestational age at birth (Fig 6B, 6D and 6F).

## Discussion

During the third trimester of fetal life, brain undergoes substantial growth, cortical folding, wiring, development of enzymatic activities, and shows the first signs of functional activity [12, 43–45]. How premature extrauterine life may affect these processes is not yet fully understood. In a previous study, we reported microstructural and metabolic differences between fetuses and preterm neonates around term, suggesting advanced brain maturation in the neonates born premature [46]. Here, we compared brain maturation in premature neonates and term neonates around term. Our findings indicate significant impact of premature transition from intrauterine to extrauterine life on brain microstructure and metabolism.

### Differences in metabolic profiles between preterm and term neonates

The part of the centrum semiovale in which the volume of interest for ^1^H-MRS was positioned was apparently not affected by any detectable ADC anomaly. At short echo-time, the main metabolic difference between the premature and term groups was the higher level of taurine in term neonates. The finding of large differences in both Y_0_ and apparent T_2_ values between PN and TN suggests that the difference in signal intensity between these groups reflects differences in taurine concentration and T_2_. Differences in metabolite T_2_ relaxation times may indicate differences in metabolite intracellular environment such as viscosity and metabolite-protein binding [46]. This aminoacid is a major osmolyte involved in cell volume regulation, neurotransmission, ion channel modulation, and neuroprotection [47–49]. Brain taurine comes from dietary intake and hepatic synthesis, but is also produced by neural cells [50, 51]. Taurine is involved in brain development, and its concentration would be the highest during synaptogenesis, before and after birth [52]. Elevated levels of taurine have been measured in the fetal rodent brain followed by a rapid decline after birth [53, 54]. Taurine is supplied to the fetal brain by the placenta and then by the maternal milk during the perinatal period [52]. The high level of brain taurine around birth is viewed as a possible protective mechanism. Kreis *et al*. found increased levels of taurine in brain with increasing GA in a population of term and preterm neonates [27]. Here, the higher level of taurine in term neonates, who have been explored in the first days following birth, could reflect this perinatal taurine peak.

At long echo time, the major metabolic differences between PN and TN were the lower Glx/H_2_O and tCr/H_2_O in preterm neonates. The Glx signal comprises both glutamate and glutamine. Glutamate is the most abundant aminoacid in the brain and the main excitatory neurotransmitter. It is involved in the glutamine-glutamate/GABA cycle between astrocytes and neurons [55]. Glutamate released by the synapse is taken up by astrocytes and converted to glutamine by glutamine synthetase. Glutamine may in turn be transferred to glutamatergic or GABAergic neurons to replenish their glutamate or GABA pools [55]. Glutamate and glutamine *de novo* synthesis and degradation are related to the tricarboxylic cycle, which produces energy in cells [55, 56]. Glutamate plays a key role in the different stages of neurogenesis during brain development and maturation including neural progenitor proliferation, migration, differentiation and survival, synaptogenesis and spinogenesis [57–59]. Glx are detectable by ^1^H-MRS in the fetal brain and increase with gestational age in the postnatal period [36, 60, 61]. While Kreis *et al*. did not find any significant differences in glutamine and glutamate concentration between preterm and term neonates around term; our results could corroborate the recent finding of reduced glutamate in the brain of preterm neonates [28].

Differences in Y_0_ and apparent T_2_ for tCr in PN and TN suggest that the finding of a reduced tCr/H_2_O in PN reflects variations in both concentration and T_2_. The lower creatine level found at a TE of 135 ms, despite the absence of any significant difference at a TE of 30 ms, could be related to alterations in the contributions of PCr and creatine to the total creatine resonance in the preterm and term neonates. Indeed, Ke *et al*. have shown bi-exponential relaxation of total creatine resonance in the adult brain as a consequence of different transverse relaxation times for methyl groups (δppm = 3.05 ppm) of PCr and creatine [62]. PCr resonance showed significantly shorter T_2_ than creatine at a field of 1.5 T (PCr: 117±21ms; creatine: 309±21 ms) [62]. The difference in tCr/H_2_O level between preterm and term neonates is observed at a TE of 135 ms, which is greater than the T_2_ of PCr. At such echo time, PCr resonance should be significantly reduced due to T_2_-weighting, whereas creatine becomes the main contributor to the tCr resonance. The difference in tCr/H_2_O between preterm and term neonates suggests that cerebral creatine level is lower in preterm neonates. Our apparent T_2_ estimate for tCr in PN is -9.23% lower than in TN, a result that could further support the hypothesis of reduced creatine contribution to the tCr resonance in PN.

Creatine and PCr play a key role in cellular energy homeostasis by replenishing ATP through the reversible transfer of a phosphoryl group from PCr to ADP upon creatine kinase activity [63, 64]. Creatine is provided by diet and endogenous synthesis, which is orchestrated by L-arginine: glycine amidinotransferase (AGAT) and guanidinoacetate methyltransferase (GAMT), two enzymes predominant in the liver and kidneys but also present in the brain [65]. AGAT catalyzes the synthesis of guanidinoacetate (GAA) from arginine and glycine, which in turn is converted to creatine upon GAMT activity [66]. Creatine supply to the fetal brain depends on maternoplacental delivery and the existence of a specific creatine receptor, CT1, expressed in the periventricular zone and in the choroid plexus, a metabolic exchange zone of the fetal brain [67, 68]. Creatine synthesis by the fetal brain, liver and kidney begins at the end of the fetal life [69] when induction of enzymatic activities involved in creatine synthesis occurs [66, 70]. Interestingly, a recent study reported a creatine deficiency in preterm neonates [71], sharing similarities with GAMT congenital deficiencies, such as decreased creatine and increased GAA urinary excretion [70]. This creatine deficit in premature neonates could be related to the early expression of AGAT during fetal development but the late near term expression of GAMT [66, 68]. In preterm neonates, enzymatic immaturity combined to renal insufficiency leading to renal leak of the amino acids involved in creatine synthesis would result in creatine deficiency [71]. A possible impairment of creatine transport in the preterm brain, as in GAMT deficiencies, is possible since GAA and creatine are competitive for the same transporter CTI [72, 73]. Creatine is crucial for brain development and functioning, and in particular for cognitive and psychomotor functions [65, 67]. Creatine deficiencies of genetic origin are responsible for severe neurological impairment including mental retardation, autism, speech delay and epilepsy [72, 74]. The same phenomenon may occur in premature neonates [72, 73].

### Differences in macromolecule content between preterm and term neonates

One of the newest findings of this study is the significantly lower level of some macromolecules in the brain of premature neonates around term. At short TE, proton spectra show the presence of mobile lipids and macromolecules whose resonances not only cause baseline distortion, but also overlap with those of metabolites and consequently affect their quantification. In this study, we used a resonance fitting procedure evaluating metabolites, mobile lipids and macromolecules simultaneously. The macromolecule signal originates from cytosolic proteins and includes numerous resonances with complex pattern. Previous high-resolution NMR studies of cytosolic fractions of human and rat brain tissue extracts allowed the assignment of some of them to the methylene protons of aminoacids in mobile polypeptide chains or to methine protons [75, 76]. Increases in brain macromolecules have been reported in multiple sclerosis [77], tumors and stroke [78, 79]. In our study, alterations in the macromolecule levels measured in the brain of preterm neonates provide additional information on the effects of premature birth on brain maturation. Our results not only show a trend for lower signal intensities of resonances common to macromolecules and mobile lipids (MM_9 and MM_12) but also a significant decrease of specific macromolecule resonances corresponding to mobile polypeptide chains rich in glutamate and glutamine (MM_27) and methine groups (MM_37 and MM-39) [75, 76]. Moreover, MM_27/H_2_O, MM_37/H_2_O and MM_39/H_2_O levels were correlated to gestational age at birth and thus directly affected by premature birth. On the other hand, MM_37/H_2_O was also positively correlated to post-conceptional age, a finding indicating that some macromolecules levels keep on increasing after the transition to extrauterine life and subsequent changes in environmental stimulation and nutrition. Our results suggest reduced protein and mobile lipid synthesis in the brain of premature neonates at term-equivalent age in comparison to term neonates.

### Study limitations

We did not have access to the urine of the neonates, and could not analyze the creatine and GAA concentrations in this fluid. Even though PN had normal clinical examination at two years of age, we cannot exclude later cognitive impairment or scholar difficulties. A clinical and MRS follow-up would be necessary to check whether these metabolic abnormalities persist later in life and represent early biomarkers for later cognitive impairment that could be used to evaluate therapeutic intervention.

## Conclusion

While we had previously reported advanced metabolic maturation in neonates born premature in comparison to fetuses around term, our current study indicates that preterm neonates, without brain injury, exhibit microstructure alterations and metabolic anomalies related to neurotransmission, energy metabolism and macromolecule synthesis in comparison to term neonates. Further studies are needed to assess whether these anomalies may persist and be involved in the frequent impaired cognitive development observed later in life.

## Supporting Information

S1 File ADC values, metabolic ratios, and macromolecule ratios. (XLSX)
